# Patients’ approaches to students’ learning at a clinical education ward-an ethnographic study

**DOI:** 10.1186/1472-6920-14-131

**Published:** 2014-07-02

**Authors:** Katri Manninen, Elisabet Welin Henriksson, Max Scheja, Charlotte Silén

**Affiliations:** 1 Department of Learning, Karolinska Institutet, Informatics, Management and Ethics, Stockholm, Sweden; 2 Department of Neurobiology, Karolinska Institutet; Care Sciences and Society, Stockholm, Sweden; 3 Department of Education, Stockholm University, Stockholm, Sweden

**Keywords:** Patient-student encounters, Clinical education ward, Patient-centeredness, Learning relationship, Attending relationship

## Abstract

**Background:**

It is well known that patients’ involvement in health care students’ learning is essential and gives students opportunities to experience clinical reasoning and practice clinical skills when interacting with patients. Students encounter patients in different contexts throughout their education. However, looking across the research providing evidence about learning related to patient-student encounters reveals a lack of knowledge about the actual learning process that occurs in encounters between patients and students. The aim of this study was to explore patient-student encounters in relation to students’ learning in a patient-centered health-care setting.

**Methods:**

An ethnographic approach was used to study the encounters between patients and students. The setting was a clinical education ward for nursing students at a university hospital with eight beds. The study included 10 observations with 11 students and 10 patients. The observer followed one or two students taking care of one patient. During the fieldwork observational and reflective notes were taken. After each observation follow-up interviews were conducted with each patient and student separately. Data were analyzed using an ethnographic approach.

**Results:**

The most striking results showed that patients took different approaches in the encounters with students. When the students managed to create a good atmosphere and a mutual relationship, the patients were active participants in the students’ learning. If the students did not manage to create a good atmosphere, the relationship became one-way and the patients were passive participants, letting the students practice on their bodies but without engaging in a dialogue with the students.

**Conclusions:**

Patient-student encounters, at a clinical education ward with a patient-centred pedagogical framework, can develop into either a learning relationship or an attending relationship. A learning relationship is based on a mutual relationship between patients and students resulting in patients actively participating in students’ learning and they both experience it as a joint action. An attending relationship is based on a one-way relationship between patients and students resulting in patients passively participating by letting students to practice on their bodies but without engaging in a learning dialogue with the students.

## Background

Patients’ involvement in health care students’ education in clinical settings is often taken for granted. Learning to take care of and to treat patients are essential parts of the education for all health-care professionals. The focus of the present study is nursing students at a clinical education ward. Since encounters with patients are common in many health-care professions, the intention is to provide knowledge about patients’ approaches to students’ learning in clinical practice, not only for nursing students, but also more generally for students studying to become health-care professionals in different fields. In research about patients’ involvement in students’ learning, both profession-specific aspects and aspects that are not tied specifically to a particular profession are highlighted. Patients can provide opportunities for students to practice clinical skills and to provide information as experts of their own illness or disability [1,2]. Patients may be real patients or actors who have been trained to simulate illness and teach or instruct students [3]. Looking across the research on patient involvement in students’ clinical training reveals a lack of knowledge about the learning processes that actually occur in encounters between health-care students’ and patients in clinical settings.

Research has shown that patients’ experiences of their own involvement in students’ learning are mainly positive and they are accustomed to the presence of students. Patients may express feelings of empowerment and self-worth by sharing experiences of illness and care and by letting students train practical skills. Helping students to learn can also give patients satisfaction [4-6]. However, not all experiences are positive. Studies have also revealed that patients may express concerns about students’ access to patient records and discussing personal matters. Patients’ negative statements are also related to encountering uncertain or disinterested students and exclusion of communication between student and supervisor [5-8].

Suikkala and Leino-Kilpi [9] found that the relationship between patients and students has an impact on students’ learning. Students can focus on either just performing tasks given by supervisors or performing care directed by the patients’ requirements and expectations. Stockhausen [10] emphasizes that learning occurs through the relationships between patient and student. So research suggests that the nature of the relationship between patient and student is important both for patients’ experiences and for students’ learning. Manninen et al. [11,12] found that a mutual relationship between patient and student constitutes the basis of students’ learning. When this mutual relationship exists, students experience external and/or internal authenticity in their learning process. External authenticity is experienced by being at a real ward and taking care of real patients. Internal authenticity refers to the experience of belonging by creating a relationship with patient and making a contribution to their care. Students’ experiences of both external and internal authenticity influence their learning process, resulting in deep learning, which is described by Marton and Booth [13].

The present study draws on a theoretical framework that allows learning to be seen as a complex phenomenon involving cognitive, socio-cultural and emotional aspects. Learning is viewed as an active construction process based on the individual’s understanding, thinking, action and interaction [14-16], involving a transformative meaning-making process and resulting in new or modified interpretations of perceptions and experiences [17]. Meaning -making here refers to a process of knowledge construction through which students make sense of their experiences in interaction with other people and the actual context in which they find themselves [14,16].

Previous research [1,2,4-6] has shown that the interaction between patients and students is pivotal in students’ learning in clinical settings. That conclusion is mainly based on interviews and questionnaires [5-7]. In order to get a deeper understanding of what actually happens in such settings we need to study the interaction between patients and students. The aim of the present study is to explore patient-student encounters in relation to students’ learning in a patient-centered health-care setting.

## Methods

### Design

This study forms part of a project exploring students’ learning at a clinical education ward from different perspectives using qualitative interpretative approaches. The present study focuses on patient-student encounters using an ethnographic approach allowing exploration of social interactions in naturalistic settings and collecting data from different sources such as observations and interviews [18,19].

### Setting

Data were collected at a clinical education ward at a department of infectious diseases at a university hospital in Sweden. The ward has eight beds training nursing students either at the beginning or at the end of their education. Fifteen students are simultaneously doing their clinical practice for six weeks. Four nurses, a nursing assistant, a clinical lecturer and a physician serve as supervisors and guarantee patient safety. A physiotherapist, an occupational therapist, a dietician and a counselor are also linked to the ward. Three supervisors (two nurses and a nursing assistant) work the morning shift and two supervisors (nurses) work the evening shift. During the night shift a nurse and a nursing assistant take care of the patients and there are no students present.

The pedagogical framework is based on patient-centered learning. The students take care of their own patients as independently as possible with support from the supervisors. They work either individually or in pairs. The students are allowed to act as nurses; they plan, perform and follow up the nursing care for their patients. The patients are informed about the organization and assured that even though the students are acting independently, the supervisors are responsible for patient safety as well as for the students’ learning.

### Participants

In the autumn of 2012 two groups of a total of 28 students completed their clinical practice at the ward. Fourteen of these students were in their second year and 14 were in their final year, and they all had previous experience of clinical practice. All 28 students were informed about the purpose of the study both orally and in writing. Patients who were Swedish- or English-speaking and did not have dementia were identified as eligible participants. The first-author contacted the eligible patients the day before the planned observations and gave them both oral and written information. All of the ten patients agreed to participate. The students who took care of these patients were also invited to participate. In all, 11 students, six in their final year and five in their second year volunteered for participation. Six of these were women (four final-year and two second-year) and five men (two final-year and three second-year), aged 21 to 36. As for the patients, there were six women and four men, aged 18 to 89.

### Data collection

Ten observations were performed by a participant observer (KM). The observer wore a nurse uniform and followed one patient and one student, except from one observation with two students, during a morning shift between 7 am and 1 pm. In all, 50 hours of observations were conducted. The observer took extensive field notes including both observational and reflective notes. The observational notes included descriptions of activities and interaction during the observation and the reflective notes included the observers’ thoughts and questions that occurred while observing. After each observation audio-recorded follow-up interviews were conducted by the observer with students and patients separately. The participants were encouraged to talk about what had happened that morning and how they felt about it. In all, 21 interviews were conducted lasting between 5–20 minutes and the interviews were transcribed verbatim. After each observation the observer transcribed the field notes, reflected on and discussed the observations with one member (CS) of the research team.

### Data analysis

The analysis followed ethnographic procedures [18,19] implying an iterative process that involves describing and examining the relationships and linkages between the data from different sources. Through such an interpretative process, an understanding of the data is generated that goes beyond the descriptive level. In an ethnographic approach the presented narratives constitute an interpretation of the actions and interactions and the intention is to transform the observations and interviews into a comprehensive text [19].

In the present study the analysis of data involved six different but interrelated steps:

1. Transcripts from the field notes and interviews were read through several times by the first author (KM).

2. Events including interaction between student and patient were marked.

3. Two of the authors (KM, CS) selected four observations for further analysis that included events observed by the observer and talked about by both students and patients. These four observations consisted of various events that were both planned and unexpected. Examples of the events are *assisting patient with different activities*, *taking vital controls* and *attending a consultation*. Two of the students were in their second year and two students in their final year.

4. Positive and negative learning situations from these events were identified. In positive learning situations both student and patient expressed positive feelings about what happened and in negative learning situations they expressed negative or not so positive feelings about what happened.

5. The learning situations were analyzed further by looking for different characteristics of these situations. Examples of these characteristics are verbal and non-verbal interactions, mutuality and non-functioning interaction.

6. These characteristics were subjected to further interpretation, resulting in two narratives consisting of themes illustrating patient-student encounters in relation to students’ learning at a clinical education ward. These themes were compared to all ten observations and no new aspects, that were not included in the themes, were found. Accordingly, it was assumed that further observations would not provide new information.

Steps four to six were conducted by KM and discussed with CS. The sixth step was discussed by the whole team (KM, CS, EWH, MS) until consensus was reached.

### Ethical considerations

The study was approved by The Regional Ethical Review Board at Karolinska Institutet in Stockholm. Participants were informed both orally and in writing about the voluntary participation and that the study would not affect student assessment or patient care and that they were able to terminate their participation at any time without any explanation. Participants were also informed about the data confidentiality. Informed written consent was obtained from all participants prior to the observations.

## Results

The results are presented as two narratives created from the data generated by the observations and interviews. The narratives consist of themes that describe patient-student encounters in relation to students’ learning in the actual context [19]. The first narrative, *Encounters between the patient and the student,* describes the interaction and the relationships between patient and student at the ward. The second narrative, *Patients’ engagement in students’ learning,* describes the two different ways that patients are engaged in students’ learning. An interpretation of the results is presented in Table 1.

**Table 1:** Patients’ approaches to students’ learning

**Learning relationship**	**Attending relationship**
• Mutual relationship	• One-way relationship
• Patient as active participant	• Patient as passive participant
• Joint action	• Training object

Patient-student encounters and interaction develop in to either a learning relationship or an attending relationship. A learning relationship is based on mutual relationship between patients and students which results in that patients are actively participating in students’ learning and they both experience it as a joint action. An attending relationship is based on one-way relationship between patients and students resulting in patient as a passive participant just letting students to practice on their bodies.

### Encounters between patients and students

Encounters between patients and students involve different types of interaction, resulting in a relationship being formed between them. The theme *Creating a good atmosphere* describes relationship-building. The nature of this relationship depends on the interaction between students and patients and is illustrated in the themes *Mutual relationship* and *One-way relationship*. Good atmosphere and dialogue leads to a mutual relationship. If the dialogue is missing, the relationship is one-way instead of mutual.

Creating a good atmosphere

The student knocks on the door, enters the room, says good morning to the patient, asks about the night and specifically about the painkillers. The patient answers that the third painkiller worked well and wonders if it might be the combination that worked. The student stands near the bed, looks the patient in the eye and explains how the different types of painkillers work and says that he will inform the doctor at the round which painkillers the patient received and how he experienced the effect of them. The patient nods his head in agreement.

[Field note, final-year male student (4), male patient (3)]

The students visit their patients and sometimes the patients are already waiting for them. If they are still asleep the students carefully wake them up. The students ask the patients how they are doing and whether they have slept well. If they have met the patient before, they also follow up on what has happed since they last met. If they meet for the first time, the students start by introducing themselves. There is a lot of smiling and laughter and the patients and the students have eye contact all the time. The patients and the students show interest in each other by asking questions and discussing not only the patients’ medical condition but also things outside the hospital world.

The students pay attention to patients as individuals…they care a lot about our well-being.

[Male patient (3)]

You can discuss your work or this and that…so we have a little bit of fun as well…

[Female patient (6)]

The students prepare the patients for the medical-technical tasks, such as taking vital controls or blood samples by giving information not only about what is going to happen, but also about how and why. The students present their plan for the shift and discuss it with the patients, to make sure that the patients understand and accept the plan. The patients are given an opportunity to ask questions and express their opinion in relation to the plan. Sometimes the patients wish to change some details and if possible the students make the changes.

The student first said that I could take the medicine later… she also checked with the supervisor…this is what I do at home if I need to go somewhere…I take the diuretics later.

[Female patient (1)]

Mutual relationship

The students show interest in their patients by obtaining information about them in different ways. They read the records and observe the patients by looking, listening and touching. They also assist the patients in their everyday activities when needed. The students put together all the information from their observations with the information from the records and reports from and discussions with peers, supervisors and physicians. Spending time together with the patients and communicating with them results in students getting to know the patients as individuals.

I got a report from my peer student, I read the nursing care plan so I knew that I knew quite a lot about the patient… I sat down and introduced myself…told her that I’d take care of her that evening and that I had noticed that she used to work as a nurse and we started to discuss…

[Second-year male student (8)]

I got the evidence for the importance of seeing the individual, listen to him and not take over…the patient is in focus and decides what we do and how and why…so he needs to be informed and understand what it’s all about.

[Second-year female student (10)]

The patients also get to know the students. They know how far the students have come in their education and how they are getting on. Sometimes the patients forget that the students are not yet graduated nurses, since the patients feel that the students have taken care of them in an equally qualified manner as graduated nurses.

It works better…nowadays they do things in the right order… take all devices with them before they come in.

[Male patient (4)]

I didn’t think of some of them as students…they showed initiative and were knowledgeable…

[Female patient (1)]

One-way relationship

When the students focus on carrying out the planned tasks, the communication between the patients and students is based on short questions and short answers about the tasks at hand. The students have few follow-up questions and these mainly relate to the effects of medicine.

It feels silly when they say they are about to put the needle in, I know that and I’m prepared…there’s no need to say that…But it would be nice to know why they take all these samples…why they need to put all those needles in …to get an explanation…My students are professional when they do things, but they don’t give me that much information or answer my questions.

[Female patient (5)]

The patients experience that the students do not enter their room spontaneously, but only when they are about to perform something or when the patients call them. The patients also express that they have to ask specifically for what they need; sometimes they even have to repeat their needs and remind the students about their wishes. The students inform the patients about the tasks they are about to perform or the patients inform the students, but there is no real dialogue between them. Both students and patients lack a holistic picture of the patients’ situation.

We didn’t have a conversation, she treated me rather than informed me…I stay silent while she works… you have done what you could and I received as much as I was able to…

[Male patient (9)]

### Patients’ engagement in students’ learning

Patients are engaged in students’ learning, but the engagement depends on the nature of the relationship between them. The themes *Patient as an active participant* and *Patient as a passive participant* illustrate the different ways of patients’ engagement.

The patient as an active participant

When the patients and the students have a mutual relationship, the patient becomes an active participant in students’ learning. The patients express an understanding of the students’ need to practise and perform by themselves, as opposed to just observing and imitating the supervisors. The patients allow the students to perform different medical-technical procedures, such as taking blood samples, vital controls and wound care. The patients also show the students where they can find a suitable vein, they advise students on how to perform different tasks and they do it together with the students and also check whether the students have all the necessary equipment before they start the procedures. The patients are willing to help the students even though the students sometimes fail and need to redo the procedure, which sometimes can even be painful for the patients.

Small mistakes happen everywhere…anyway he knew what he was doing…he was like a professional. It is normal that you make some minor mistakes.

[Male patient (3)]

Not only do the patients let the students perform these medical-technical procedures; they also give the students information about themselves. They tell the students how the illness affects their lives. So the patients actively take part in students’ learning by allowing them to practise, telling about themselves, showing, giving advice, holding things and reminding them. The patients also express awareness of their participation in students’ learning.

I think it’s fantastic that she shared her story with me…I feel warm inside and it inspires me to become a nurse.

[Final-year female student (3)]

They always ask if it’s okay before they proceed with something. They need to practice as much as possible. One day they‘ll be responsible for everything and it’s good to have had the support and the possibility to practice. Sometimes I even give them some advice on how they should do something and assist by holding things.

[Female patient (7)]

Patients also express that they gain knowledge about their own condition when they communicate with students.

I learnt something today…about my blood pressure… I thought that I had medicine for that but it turned out I didn’t. They [the students] listen to me and they explain as good as they can and even better… that is different from other wards where they [nurses and physicians] think that because they know something everybody knows it.

[Female patient (2)]

The patient as a passive participant

If the mutual relationship between patients and students is missing, the patients remain passive participants in the students’ learning. This means that the patients still have an understanding of the students’ need to practice and they are willing to help the students by allowing the students to train but do not actively engage in a dialogue with students around the health-care procedures The students focus on performing medical-technical tasks and inform patients of what will happen and how, but not always explain why. The verbal communication between the patients and the students is curt. When the students are performing medical-technical tasks, the patients often turn their heads away and patients and students generally have quite little eye contact with one another.

The student informs the patient that he will take some blood samples and asks if she bled from the nose or vomited during the night. The patient says none of these things happened. They talk quietly and briefly. The student looks for veins in the patient’s arm. The patient turns her head away. The student inserts the needle into a vein but does not succeed. He tells the patient, who looks towards the student for a short while and then turns her head away again without saying anything. The student continues to look for veins and says that it is difficult since the patient is dehydrated. He finds a vein, inserts the needle and asks the patient if it hurts. The patient says that it hurts. The student does not respond to that. He does not succeed this time either and says that he will wait a while and asks the patient if it is okay that he takes the vital controls instead. The patient nods her head.

[Field note, final-year male student (6), female patient (5)]

As a passive participant the patients help the students by letting them practice. The patients offer parts of their bodies for students to practice on. The students express that they just repeat tasks they already know how to perform rather than learn something new from the patients.

To be honest, I didn’t learn much from her…she was doing well…I was checking the blood pressure…it was good to practice that.

[Second-year female student (11)]

The patient-student encounters in relation to students’ learning at a clinical education ward are illustrated in Table 1. The encounters result in a relationship which can be either one-way or mutual. The relationship thus becomes an attending relationship with patients as passive participants, or a learning relationship with the patient being involved as an active participant in students’ learning.

## Discussion

Good atmosphere and mutual relationships are of importance for patients’ participation in students’ learning process as active participants. In the present study, the patients are engaged in students’ learning but differ in the extent to which they are active participants or just letting the students practice on their bodies. Patients’ active participation can develop into a learning relationship providing rich opportunities for students to learn from and with the patients.

Monrouxe et al. [20] found that patients usually participate passively as objects rather than as subjects in students’ learning. But what are the the prerequisites for patients’ active participation and for achieving a mutual relationship? Based on the research presented here we suggest that a pedagogical framework emphasizing patient-centeredness and student responsibility offers genuine opportunities for students to create a mutual relationship with patients. The patient-centered pedagogical framework provides the students with opportunities to take an interest in patients’ situations, interact with them and establish an ongoing dialogue, since they spend time together continuously. This conclusion is in line with Warmington [21], who points out attentiveness, respectful dialogue and commitment as important elements in patient encounters. Debyser et al. [4] also stress the patients’ appreciation for students spending time with them. This study shows that when the students are interested in patients as individuals and subjects, not solely as objects on which to practice, they manage to create a good atmosphere. Similarly, the patients become interested in the students and are willing to help them to learn. This interaction between patients and students can result in a mutual relationship.

When students and patients work together they also engage in a meaning-making process that potentially results in transformative learning and knowledge construction [14,16,17]. When students assume a holistic approach to the patients’ situation, the encounters become meaningful for both of them. By relating experiences with the patients to their previous knowledge, the students enhance their understanding and readiness for future encounters. They construct this new understanding based on the encounters with patients as active participants. The patients gain knowledge and experience by contributing to students’ learning. In Manninen et al. [11] students expressed that they learned *from, through and with* the patients. The present study shows that when students are patient-centered the learning becomes a joint action where patients are active participants and the mutual relationship develops into a learning relationship as presented in Table 1.

Moreover, patient-centeredness consists of students’ taking care of their own patients with the support from the supervisors when needed. Since the students are encouraged to work independently, the patients and the students interact on their own, without the supervisors being present. The patients and the students establish a continuous dialogue and the patients know what is going on and why and also who is doing what. The students are informed of the patients’ situation concerning medical and nursing care and both of them get a holistic picture of the patients’ situation. Accordingly, the patient-centeredness enables patients to participate in the students’ learning in a direct and active way, although they are not specifically trained for this participation. Bleakley and Bligh [22] as well as Lauckner et al. [8] emphasize the need for patients’ participation and students’ learning *with* patients. The present study illustrates how this can be achieved.

Despite the pedagogical framework not all students are patient-centred and not all manage to build mutual relationships. When the students do not spend sufficient time with the patients the interaction will be scarce and the dialogue will be lacking. This results in an attending relationship where the patient takes the role of a passive participant still willing to let the students practice, but as an object rather than a subject, see Table 1. In such circumstances, the students do not show any particular interest in the patients’ situation; they are focused on performing tasks and so get bits and pieces of information without seeing how the pieces can be linked together, see also Manninen et al. [12]. This means learning *from* the patients rather than *with* the patients as emphasized by McLahlan et al. [5] and Towle and Godolphin [2]. Hence, the supervisors can play a crucial role in supporting students in integrating theoretical knowledge and patient information in nursing care. Accordingly, the results of the present study will be used to further develop the supervision at the clinical education ward. Supervisors are important for students’ learning and more research is needed on how supervisors can encourage and stimulate students to create mutual relationships in this kind of setting. The present study focuses on understanding students’ learning processes in relation to patients’ approaches. In order to illuminate the learning outcomes in relation to patients’ approaches, further research could be directed towards studies comparing learning outcomes in settings that specifically enhance patient-centered care to learning outcomes in other clinical settings.

### Methodological considerations

In the preset study participation was voluntary both regarding patients and students and therefore it is possible that not all kind of experiences have been captured. However, the participants, both patients and students, expressed diverse experiences concerning the encounters between them, which was the issue at hand. Inviting students from different levels was an attempt to get varied experiences [23]. In an ethnographic approach the participant observer is actively engaged with the participants and therefore reflexivity is needed. During the fieldwork reflexivity was ensured by taking reflective notes and discussing the observations [19]. The first author had a pre-understanding of the setting which has been challenged continuously within the research group. Moreover, investigator triangulation was used to increase trustworthiness. To allow transferability of the results the setting for the study is described at some length [23,24]. Relating the results to the learning theories [14-17] attempts to enhance the applicability of the results in other contexts [19,23].

## Conclusions

When the patient-student encounters at the clinical education ward result in a mutual relationship the patients become active participants in students’ learning. A patient-centered pedagogical framework enhances learning as a joint action where a learning relationship between the patients and the students can be established. Providing students with possibilities to interact with patients in a meaning making learning process could be regarded as a pedagogical strategy to enhance learning for students within all health care professions. The supervision should aim to support the students to create mutual relationships with patients enhancing holistic understanding of the patients’ situation. The characteristics of a learning relationship and an attending relationship in patient-student encounters inherit crucial aspects that are most likely transferable to other health-care professions. However, to transfer these results to other settings, critical reflection on what these crucial aspects mean in relation to different professions and different health-care settings will be needed.

## Competing interests

The authors declare they have no competing interests.

## Authors’ contributions

KM designed the study, participated in data collection and data analysis and drafted the manuscript. CS participated in study design, data analysis, helped to draft and critically revised the manuscript. EWH and MS made important contributions to the study design, discussed the results and critically revised the manuscript. All authors read and approved the final manuscript.

## Pre-publication history

The pre-publication history for this paper can be accessed here:

http://www.biomedcentral.com/1472-6920/14/131/prepub
